# Disease Conditions and Health Information Needs Among People Who Inject Drugs: Engendering Research to Policy and Interventions Initiatives

**DOI:** 10.3390/ijerph22030340

**Published:** 2025-02-25

**Authors:** Chris Chukwunyere Njoku, Judith Ifunanya Ani, Lucky Norah Katende-Kyenda

**Affiliations:** 1 Inspire World International Foundation, Abuja 961102, Nigeria; chrischukwunyere@gmail.com; 2 Faculty of Health Sciences, Walter Sisulu University, Mthatha 5117, South Africa; nkatende-kyenda@wsu.ac.za

**Keywords:** people who inject drugs (PWIDs), health information needs, public health interventions, disease conditions, qualitative research

## Abstract

Background: Injecting drug use is a global public health challenge with multifaceted consequences, not only for people who inject drugs (PWIDs) but also for society at large. Their vulnerability necessitates a deeper exploration of their health information needs, aiming to leverage evidence-based research to shape effective interventions for their well-being. Method: This study employed a qualitative method to gain insights into disease conditions and health information needs of PWIDs. Through purposive and snowball sampling, 71 in-depth interviews were conducted and thematically analyzed. Results: This study included 43 males and 28 females, predominantly aged 26–35 (59.2%), who had low socioeconomic status. The most reported disease conditions varied and included malaria, infections, and diabetes. Findings revealed a complex understanding of their disease conditions and management practices. Participants emphasized a critical need for access to reliable and comprehensive health information, while also highlighting the significant barriers they face in obtaining this information. Additionally, their preference for receiving health information in video formats, written articles, and through outreach programs underscored their desire for knowledge to make informed decisions. As co-creators and stakeholders in their health, participants expressed a clear demand for sustainable and free healthcare, mosquito nets, and regular outreach programs. Conclusions: While drug use presents a significant public health issue, effective interventions for PWIDs require a multifaceted approach that begins with understanding their perspectives and actively involving them as co-creators of their health solutions. Abandoning this population contradicts the Sustainable Development Goals’ mandate to ensure no one is left behind. Thus, all stakeholders must prioritize inclusive and participatory approaches to address the complex health information needs of PWIDs.

## 1. Introduction

Drug use is a global public health and social problem [1,2], posing a significant threat to the well-being of the individuals involved, their families, as well as society at large. This multilevel impact of drug use encompasses social, economic, and health dimensions. In the context of drug use in Nigeria, the landscape presents a significant and complex challenge. The United Nations Office on Drugs and Crime [3] statistics revealed that approximately one in seven individuals, aged 15 to 64 years, has engaged in nonmedical drug use within the past year, excluding tobacco and alcohol, the highest levels concentrated among individuals aged 25 to 39 years. This striking prevalence of any drug use is estimated at 14.4 percent, translating to 14.3 million people who have engaged in the consumption of psychoactive substances for nonmedical purposes in the past year. Within a gendered context, it is evident that, among every four drug users in Nigeria, one is a female. Approximately three quarters of drug users in Nigeria are predominantly located in rural areas, with one notable exception being the south-west zone, where three quarters of drug users are situated in urban areas [3]. The north-central zone exhibits an estimated annual prevalence of 10 percent, amounting to approximately 1.5 million individuals. Within this zone, Plateau State, which serves as the study setting, stands out with the highest estimated prevalence, representing a substantial population of over 240,000 individuals engaged in drug abuse.

Globally, injecting drug use (IDU) occurs in 179 of 206 countries, with 17.8% of the 15.6 million people who inject drugs living with diseases including HIV and hepatitis C (HCV). This widespread issue has a profound impact on global public health, leading to higher rates of disability, illness, and death [4,5,6]. IDU carries a significant burden of harm and various serious drug-related consequences [7]. This includes the transmission of life-threatening conditions such as HIV and viral hepatitis, fatal and nonfatal overdoses, as well as bacterial infections occurring at injection sites [8]. Scholars [9,10,11,12] further underscored the severe health risks associated with IDU, including infections and damage to skin, tissue, and blood vessels, which result in increased hospitalizations, morbidity, and mortality. A finding [11] documented the alarming rise in hospitalizations among young people who injected drugs in the United States, placing them at heightened risk of developing infective endocarditis (IE)—a serious heart infection associated with high morbidity and mortality.

In Nigeria [3], it was estimated that about 21 percent of high-risk drug users, totaling an estimated 80,000 individuals, have engaged in injecting drugs use in the past 12 months that preceded the study. It is essential to note that this issue is not limited to males, as nearly 17 percent of those who injected drugs were females. Over 50 percent of these PWIDs reported using them daily or nearly daily. Of particular significance was the gender-related aspect, as women were slightly more inclined than men to participate in this risky behavior. When it comes to the locations of drug injection, PWIDs predominantly injected drugs in familiar and often risk-laden settings, including in their own place of residence, a drug dealer’s premises, or the residence of their sexual partner, in the company of friends, acquaintances, spouses, or individuals they knew in general [3].

The importance of providing targeted health information to (PWIDs) to mitigate the associated health risks has been emphasized [9]. In line with this, Scholars [11] advocated for evidence-based harm reduction strategies beginning with an acknowledgment of the significant health risks of injection drug use, including its contributions to increased morbidity and mortality, and the need for health information for PWIDs.

Scholars [13] further highlighted the dearth of research on the health information needs of people who inject drugs, noting that most studies were conducted from the researchers’ perspectives rather than those of PWIDs themselves. To this end, available knowledge on what health information PWIDs have access to was examined, including their specific needs, how they engaged with information, and the most effective methods of dissemination. However, this study and similar others [14,15] were conducted in the United States, leaving gaps in understanding the context of other countries like Nigeria, where IDU remains under-researched. Thus, the necessity for more studies and developing comprehensive health information resources tailored to the needs of PWIDs was underscored. Also, the importance of exploring the health information needs of PWIDs from their own perspectives was emphasized by these scholars. Hence, this study adopted an insider perspective where participants were seen as co-researchers and co-creators.

In Nigeria, studies on drug use are not only sparse but predominantly focused on prevalence and correlational studies [16,17,18]. There is little up-to-date evidence on the health needs of people who inject drugs. A study [19] emphasized the need for PWIDs to access comprehensive health information to address multiple health challenges, including mental health issues and chronic illnesses. This study builds on such insights, aiming to illuminate the under-researched area of health information need among PWIDs in Nigeria. This study, amongst its objectives, advocates for the prioritization of the voices and experiences of PWIDs to better inform targeted and effective health interventions. To the best of our knowledge, this is one of the few qualitative studies that assess disease conditions and health information needs of PWIDs in the selected north-central Nigerian community. Therefore, this study seeks to answer the question: What are the disease conditions and health information needs of PWIDs in the selected north-central Nigerian community?

## 2. Materials and Methods

### 2.1. Research Design

This study assessed health information needs among people who inject drugs (PWIDs) in Plateau State, Nigeria, purposively selected. The research aimed to gather qualitative insights to identify disease conditions and the health information needs of PWIDs to inform targeted interventions. The qualitative design allowed for a thorough exploration of their experiences, perceptions, and challenges, yielding valuable insights into their unique health information needs. In-depth interviews were utilized to gather rich and detailed data from the participants, who are PWIDs. These methods enabled the researchers to delve deeper into their subjective experiences and perspectives. By employing a qualitative research design, this study went beyond surface-level descriptions to explore the social and contextual factors that influence their health status and health information needs. Also, this design provided an opportunity to capture the voices and narratives of these participants, shedding light on their unique challenges and opportunities for interventions.

### 2.2. Sampling Technique

For participant selection in this study, a two-step sampling approach was utilized. Initially, a purposive sampling technique was employed to select participants, who are PWIDs in Plateau State, irrespective of their gender and duration of drug use. Purposive sampling allowed for the targeted inclusion of individuals with firsthand experience and insights into this specific population under study. This approach ensured that participants possessed the necessary knowledge and perspectives to contribute meaningfully to the research objective.

To augment the sample and reach out to PWIDs who might be challenging to locate or hesitant to disclose their drug use, the snowball sampling technique was employed too. Existing participants who had been selected through purposive sampling were asked to refer other PWIDs they knew. This approach created a chain referral network that enabled the researchers to access a broader range of PWIDs who might not have been initially identified through the purposive sampling process.

### 2.3. Data Collection

A preliminary field observation in drug use hotspots was conducted first. This involved physically visiting and observing locations where drug use activities were known to occur. Through these field observations, contextual information was gathered, which provided insights into the environment, behaviors, and dynamics related to drug use. In addition to the semi-structured interviews, field notes were collected during the preliminary observations of drug use hotspots. This provided contextual insights into the environment in which participants were situated, allowing researchers to better understand the setting and the challenges faced by PWIDs. This on-the-ground data collection approach helped to capture the real-life experiences of PWIDs and provided valuable contextual information for the study.

Following the field observations, face-to-face, semi-structured, in-depth interviews were conducted with the selected PWIDs. These interviews served as a primary data collection method and allowed for a deeper exploration of the participants’ experiences and perspectives. The interviews were conducted in a private and comfortable setting, promoting open and honest discussions.

The semi-structured nature of the interviews provided a flexible framework while ensuring key topics were covered consistently across participants. The interviews specifically focused on capturing information related to prevalent disease conditions, health-seeking behaviors, barriers to accessing healthcare, and the health information needs of the PWIDs. Probing questions were used to encourage participants to elaborate on their experiences and provide detailed insights.

By combining field observations with in-depth interviews, this approach aimed to provide a comprehensive and nuanced understanding of the health information needs among PWIDs in Plateau State, Nigeria. It also strengthened the validity and richness of the study’s findings.

### 2.4. Study Participants

For this study, a total of 71 PWIDs were recruited using a combination of purposive and snowball sampling approaches. Given that the population under study represents a special and hard-to-reach population, the sample size of 71 participants was determined through purposive and snowball sampling to ensure a diverse and representative subset of the population. The adequacy of the sample was confirmed during data analysis, as data saturation was reached when no new themes or insights emerged from additional interviews [20]. The purposive sampling approach was initially employed to identify and select people who inject drugs (PWIDs) who met the inclusion criteria. This method allowed for a targeted selection of participants who could provide valuable insights for the study. Additionally, the snowball sampling approach was used to expand the sample and reach individuals who might be more difficult to locate or hesitant to disclose their drug use.

### 2.5. Inclusion Criteria

The inclusion criteria for participant selection were individuals who have a history of injecting drugs, regardless of their gender or duration of drug use.

### 2.6. Interview Guide

To ensure a systematic and comprehensive data collection process, a semi-structured interview guide was utilized. The guide served as a framework for the interviews while allowing flexibility to explore emerging themes and concepts. It consisted of open-ended questions specifically designed to elicit detailed information about the experiences, perceptions, and opinions of people who inject drugs (PWIDs) regarding disease prevalence in their community and their health information needs.

Before the main data collection, a pretest was conducted with a small group of PWIDs. This pre-test aimed to evaluate the clarity and comprehensibility of the interview guide. Feedback from the pre-test was collected and used to refine the interview guide, ensuring that the questions were clear, concise, and capable of eliciting in-depth responses from the study participants. The final interview guide was structured in a logical sequence, starting with introductory questions to establish rapport with the participants. It then progressed to explore their experiences and knowledge of prevalent diseases in their community. The guide also included questions about their access to healthcare services, their perceptions of the barriers and challenges they face, and their health information needs. These questions allowed participants to provide detailed narratives, share personal experiences, and express their unique perspectives. Probing questions were used to encourage participants to delve deeper into their thoughts and provide additional information when necessary.

### 2.7. Data Analysis

The data collected for this study, including interview transcripts and field notes, were subjected to rigorous analysis using inductive thematic analysis, which is grounded in a realist perspective. This approach allowed us to identify patterns within the data without imposing preconceived theoretical frameworks. Thematic analysis is a widely used qualitative data analysis method that involves identifying, organizing, and interpreting patterns, themes, and key issues within the data. During the analysis process, the researcher immersed themselves in the data, familiarizing themselves with the content and identifying initial categories that captured the essence of the participants’ experiences and perspectives. These were then organized into potential themes, which were refined and reviewed iteratively to ensure accuracy and coherence. Field notes were reviewed in conjunction with the interview data to enhance the depth of the thematic analysis. The observational data provided additional context to the participants’ narratives, helping to contextualize the interviews. By integrating these two data sources, the analysis provided a richer understanding of the people who inject drugs (PWIDs) in this study. Through a systematic and iterative process of theme development, common patterns, recurring ideas, and significant findings related to disease conditions and health information needs among PWIDs emerged from the data. The analysis facilitated a deep understanding of the participants’ experiences and needs.

Furthermore, interviews were audio-recorded with participants’ consent and transcribed verbatim. Transcripts were translated into English. During translation, the primary focus was on preserving the core meaning of participants’ responses rather than maintaining exact phrasing. While care was taken to reflect participants’ natural speaking style, some translations inevitably resulted in more structured and formal wording to ensure clarity and coherence in a written format.

### 2.8. Trustworthiness and Rigor

Several measures were taken to ensure the trustworthiness and rigor of this study, including conducting member checking, engaging in regular peer debriefing sessions, seeking external audit, implementing a clear research design, collecting detailed data, and conducting thorough data analysis, to enhance the study’s findings.

Firstly, member checking was conducted, which involved providing participants with the opportunity to review and provide feedback on the data analyzed. This process enhanced the credibility and confirmability of the findings as it allowed participants to validate the interpretation of their experiences and ensure their perspectives were accurately represented. Secondly, regular peer debriefing sessions were held within the research team. These sessions facilitated discussions on interpretation, consistency, and potential biases. Furthermore, an independent external audit was conducted to assess the research process, data analysis, and findings. Independent auditors, who were not involved in the study, reviewed the methodology and data to ensure objectivity and rigor. Data shared with the auditors was anonymized and deidentified to protect participant confidentiality, and informed consent was obtained for this external review. The external audit, approved by the Ethics Review Board, contributed to the validation of the study’s methodology, enhancing the overall validity and reliability of the findings.

Participants’ selection was based on predetermined criteria, ensuring representation and diversity within the sample. During data collection, detailed notes and recordings were taken to ensure accurate representation of participants’ perspectives. Thorough and systematic data analysis was conducted, involving organizing and categorizing data, identifying patterns and themes, and employing appropriate analytical frameworks. This process ensured transparency, consistency, and a comprehensive understanding of the prevalent disease conditions and health information needs among people who inject drugs (PWIDs).

### 2.9. Ethical Considerations

To ensure ethical conduct of this study, several measures were implemented to protect the rights and well-being of the participants. Ethical approval was obtained from the National Health Research Ethics Committee (NHREC), with the approval number NHREC/01/01/2007-11/05/2023. The NHREC is a regulatory body in Nigeria that oversees research ethics and ensures compliance with ethical principles and standards. Prior to data collection, informed consent was obtained from all participants. The informed consent process involved providing detailed information about the study, its purpose, potential risks and benefits, and the voluntary nature of participation. Participants were fully informed of their rights, including the right to withdraw from the study at any time without consequences. The consent process emphasized the importance of maintaining confidentiality and the privacy of participants’ information. To protect the participants’ identities and maintain confidentiality, strict measures were implemented. Participant identities were anonymized, and any identifying information was removed from the data collected. By ensuring anonymity, participants’ privacy and confidentiality were safeguarded.

## 3. Results

In Table 1 below, participants’ background information is presented. Slightly over half (52.5%) of the participants were male. The majority were between the ages of 18 and 37, underscoring young people in injecting drug use (IDU). The majority (66.2%) were single and over 5 in every 10 (52.1%) had only secondary education. Most of them (43.7%) were artisans/self-employed and the majority (28.2%) earned between 1000 and 10,000.00, all indicating a low socioeconomic status.

These findings corroborate a study [21] which stated that people who inject drugs (PWIDs) are particularly vulnerable due to characteristics such as lower education levels, being single, and low monthly income. The demographic data from the current study align with these conclusions, as the majority of participants were young, single, had only secondary education compared to tertiary, and earned low incomes.

### 3.1. Experiences of Living with Diseases and Perceptions Among People Who Inject Drugs (PWIDs)

Analysis revealed that PWIDs experienced different health conditions that included malaria, typhoid, infection, hypertension, and diabetes. Participants described these diseases, their experiences, and how they affected them.

Accordingly:

*Malaria is a constant struggle for us. The mosquito-infested areas where we gather make us vulnerable to the disease. We don’t have proper protection against mosquitoes, and it’s challenging to find clean and safe places to sleep. Many of us have experienced recurring malaria, and it takes a toll on our health and daily lives. We often have to endure fever, cold, and body pains, which makes it difficult to continue with our activities. It’s frustrating because we know that preventing malaria is important, but the conditions we live in make it hard to escape this constant threat.*

(PWID/Male/27 years)

This participant highlighted the significant burden of malaria. The quote underscores the challenges faced by PWIDs in terms of living in mosquito-infested areas and lacking proper protection against mosquitoes. The participant’s experience also sheds light on the recurring bouts of malaria and the negative impact it has on their health and daily activities. It also emphasizes the frustration of knowing the importance of malaria prevention but facing difficulties in escaping the constant threat due to the challenging living conditions. As studies have noted, people who inject drugs encounter a variety of risks at multiple levels, including environmental factors, which can lead to negative health outcomes [22]. Previous research [23] also highlighted malaria outbreaks among PWIDs, despite the fact that malaria is rarely transmitted through needle sharing in this population.

Highlighting another disease, the frequent outbreaks of typhoid among PWIDs in Plateau State, Nigeria, draw attention to the challenges they face living in crowded and unsanitary conditions, where access to clean water and proper hygiene practices are limited.

Thus:

*Typhoid outbreak is common in our community, especially among those of us living in crowded and unsanitary conditions. We often face challenges in maintaining hygiene and accessing good drinking water, which increases our chances of getting typhoid. The high fever, stomach pain, and tiredness that come with typhoid can be painful and affect us and our wellbeing.*

(PWID/Male/32 years)

The experiences shared by the participants also reflected the presence of hypertension among the PWIDs. For example, this participant said:

*Some of us have high blood pressure, maybe because we stress ourselves too much. The constant worries about our safety, financial struggles, and lack contribute to our hypertension. It is challenging to manage our blood pressure levels while dealing with the realities of our drug use and the stigma associated with it. Access to regular healthcare and medications is limited for us, which adds to the difficulty of managing hypertension. We are often stressed, and we have health problems which affects our overall well-being.*

(PWID/Female/39 years)

The participant’s account of hypertension within the PWIDs community echoes broader concerns regarding substance abuse and its role in cardiovascular health. Like how illicit drugs like cocaine, marijuana, and amphetamines contribute to elevated blood pressure [24], the stressors experienced by PWIDs—ranging from financial instability and safety concerns to the stigma of drug use—further compound their vulnerability to hypertension. The participant’s lived experience reinforces the finding that managing hypertension in this population is especially challenging, as the combination of drug-induced risks and environmental stressors places significant strain on their cardiovascular health. This cyclical relationship between stress, substance abuse, and limited healthcare access highlights the critical need for targeted interventions that address not only the direct effects of drug use on blood pressure but also the underlying social determinants that exacerbate these health conditions among PWIDs.

Diabetes also emerged as another significant health concern among this community. Thus:

*Living with diabetes as an injecting drug user brings its own set of challenges. We struggle to maintain a healthy lifestyle and adhere to medications while dealing with our drug use. Access to regular healthcare and diabetes management resources is limited, making it difficult to monitor our blood sugar levels and prevent complications. The lack of education and support to manage our diabetes increases the burden we carry. It is difficult trying to balance managing our health with the struggle of our circumstances as drug users.*

(PWID/Male/48 years)

The participant’s account highlights the profound challenges faced by PWIDs living with diabetes, particularly in the context of Plateau State, Nigeria. This experience mirrors broader findings in the substance abuse literature, which point to an elevated risk of metabolic syndrome and diabetes among individuals who misuse drugs [25]. The damage caused by substance abuse, including disruptions in glucose homeostasis, places these individuals at greater health risk compared to others. As the participant explained, managing diabetes while struggling with drug use creates a “balancing act”, where limited access to healthcare and a lack of tailored support makes it nearly impossible to maintain stable blood sugar levels and prevent complications. The additional strain of navigating diabetes in an already vulnerable group not only heightens health risks but also emphasizes the importance of providing targeted education and diabetes management resources for PWIDs. Addressing these multifaceted challenges is essential for improving health outcomes and mitigating the compounded effects of substance abuse and chronic conditions like diabetes.

Participants’ accounts of “toilet infection”, a colloquial term for sexually transmitted infections (STIs), highlight the prevalence of these infections within the PWIDs community, driven by high-risk behaviors such as unprotected sexual activity and needle sharing. Thus:

*Toilet infection is a prevalent concern among us. Due to our situation as drug users (you understand) and engagement in unprotected sexual activities among many here, we are particularly vulnerable to having STIs. We often face lack of access to proper healthcare services and information which increases the problem, as many of us are unaware of the importance of regular testing and treatment. The stigma surrounding STIs further hinders our ability to seek help and support. It is a constant worry and reminder of the risks we face and the need for comprehensive sexual health education and accessible healthcare services.*

(PWID/Female/28 years)

The above aligns with broader research indicating that drug use and rising STD cases represent co-occurring epidemics, with PWIDs being at an elevated risk of acquiring STIs [21]. The limited access to healthcare services, compounded by a lack of awareness and the stigma surrounding STIs, exacerbates the vulnerability of PWIDs to these infections. As the participant shared, their constant worry about contracting STIs, combined with inadequate healthcare access and the stigma they face, makes managing these health risks even more challenging. This, therefore, underscores the urgent need for targeted interventions that not only address high-risk behaviors and infectious diseases like STIs but also tackle the broader health challenges faced by PWIDs, such as noncommunicable diseases like hypertension and diabetes. Comprehensive sexual health education and accessible healthcare services must be prioritized to mitigate the co-occurring risks associated with substance use and STIs, ultimately improving health outcomes and the overall well-being of this vulnerable population.

While the experiences of these diseases were widely acknowledged by the PWIDs, their perceptions toward these health conditions provided deeper insights into their experiences. Their beliefs and feelings about the diseases varied, revealing a range of perspectives that went beyond the mere recognition of their occurrence. Overall, they believed they were caused by “*Dirty environment*”, “*Lack of nets*”, “*Mosquitoes*”, “*Unclean environment*”, “*Smoking*”, “*Lack of access to clean water*”, “*Unprotected sex*”, “*Sharing of personal belongings*”, and “*Stress*”. These multifactorial beliefs held by people who inject drugs (PWIDs) reflect their understanding within their own context. They perceive the influence of environmental conditions, mosquito-borne diseases, unclean surroundings, and personal behaviors like smoking as these multifactorial factors. Understanding these can inform targeted interventions and health education programs to address misconceptions, promote awareness, and implement preventive measures to reduce the burden of diseases among these PWIDs.

### 3.2. Barriers to Accessing Health Services

Analysis revealed several key barriers they face when accessing healthcare. These challenges emerged as follows:

“Lack of access to free healthcare”;
“Difficulty in accessing doctors”;
“Lack of health centers close by”;
“Lack of data to access health information online”;
“Lack of funds”;
“Lack of medication”;
“Lack of good living facilities like toilets”.

Impliedly, the participants expressed difficulties in accessing affordable or free healthcare services, which hindered their ability to receive necessary medical attention and treatment. This highlights how different groups in society often face unequal opportunities to achieve good health. These differences are not random; they are shaped by systemic factors such as access to healthcare, education, income, and living conditions [26,27,28]. As a result, some groups experience unfair and avoidable disadvantages in their health outcomes compared to others. Addressing these disparities requires understanding and tackling the underlying social, economic, and structural barriers that limit certain groups’ ability to achieve optimal health.

*One of the main challenges we face is the lack of access to free healthcare services, which makes it difficult for us to receive the medical care we need without incurring too many expenses.*

(PWID/Male/45 years)

This suggests that there is a barrier preventing the participants from receiving necessary healthcare services, perhaps due to the financial burden, typically out-of-pocket payments, associated with accessing healthcare. Typically, in Nigeria, most people pay for healthcare directly out of their own pockets, a system known as out-of-pocket (OOP) payments [29]. This system creates significant challenges, particularly for vulnerable populations like PWIDs, who often have limited financial resources and face stigma when accessing healthcare services. For PWIDs, the high reliance on OOP payments can mean that they delay or avoid seeking necessary medical care, further exacerbating their health risks and vulnerabilities. To address this issue, there is an urgent need to reduce dependence on OOP payments by introducing alternative healthcare financing mechanisms, such as affordable health insurance and government-subsidized programs. For PWIDs, targeted interventions like subsidized care, harm reduction programs, and stigma-free healthcare services are crucial to ensure equitable access and improve health outcomes within this high-risk group.

Participants also faced obstacles in scheduling appointments and accessing healthcare professionals. This limited access to doctors and healthcare providers affected their ability to receive timely and appropriate medical care, highlighting the need for improved healthcare system efficiency, reduced wait times, and better co-ordination between patients and healthcare providers.

*Accessing doctors and healthcare professionals is a struggle for many of us. It can be challenging to schedule appointments and get the necessary medical attention in a timely manner….*

(PWID/Female/51 years)

The absence of nearby health centers and medical facilities posed significant challenges, resulting in longer travel times and increased inconvenience when seeking medical assistance.

*…Living in areas with a shortage of health centers and medical facilities poses a significant challenge. We often have to travel long distances to receive basic medical care….*

(PWID/Male/56 years)

This challenge suggests that individuals have limited or no proximity to healthcare facilities in their communities. It underscores the importance of establishing accessible healthcare infrastructure, especially in underserved areas, to ensure that people can receive essential medical services without excessive travel or inconvenience.

The lack of reliable internet access and data as a barrier to accessing health information online was also highlighted. Limited connectivity prevented them from utilizing online resources to obtain relevant health information and guidance.

Accordingly:

*Limited internet access and the lack of available data prevent us from accessing health information online. We miss out on valuable resources and guidance that could help us know more and make good healthcare decisions…*

(PWID/Female/23 years)

This implies that the PWIDs face limitations in accessing valuable health information online due to limited internet access or insufficient (or lack of) data plans. It emphasizes the importance of expanding internet connectivity, finding ways to support them with data plans, internet access, and digital health literacy promotion to empower individuals with reliable and accessible health resources. A study [30] highlighted the growing influence of online health information on patient behavior and healthcare decision-making. E-health services, such as online appointment scheduling and practice-specific websites, which significantly affect patients’ choice of healthcare providers. Additionally, nearly half of health internet users in the study [30] reported changing their diet and increasing physical activity based on health information found online, while 45% were prompted to schedule medical appointments and 40% sought clarification on diagnoses and treatments during consultations. These findings underscore the transformative potential of internet access in improving health decisions and encouraging proactive healthcare behavior. For vulnerable populations like PWIDs, access to the internet and reliable online health information could empower them to make informed decisions about their health, seek timely medical care, and adopt healthier lifestyles, thereby addressing some of their unique healthcare challenges.

Financial constraints also emerged as a significant challenge, preventing participants from affording necessary healthcare services, medications, and treatments because these are typically out-of-pocket expenditures. The lack of financial resources further exacerbated their difficulties in accessing adequate healthcare. It therefore highlights the need for financial assistance programs, insurance coverage, or other mechanisms to alleviate the burden of healthcare costs, ensuring that individuals can afford necessary treatments and services.

*…Financial constraints are a major obstacle for us in accessing healthcare. The lack of financial support prevents us from affording necessary treatments, medications, and even basic healthcare services….*

(PWID/Female/34 years)

Concerns about the limited availability of medications, which impeded PWIDs’ ability to manage chronic conditions and address their healthcare needs effectively, were stated. This suggests that they experience difficulties in accessing essential medications, which can compromise their ability to manage disease conditions and treat them effectively. It underscores, too, the importance of reliable medication supply chains, affordable prescriptions, and efforts to address medication shortages.

*…The scarcity of essential medications is a big problem to us. It becomes difficult for us to manage chronic conditions and treat illnesses effectively due to the unavailability of necessary medications….*

(PWID/Male/52 years)

Finally, the absence of proper sanitation facilities, including access to clean toilets, posed health risks and hampered efforts to maintain hygiene and prevent the spread of diseases within their communities. Inadequate sanitation facilities, which can contribute to health risks and the spread of diseases, was implied, emphasizing the need for improved sanitation infrastructure, access to clean water, and hygiene education to ensure a safe and healthy living environment.

*…The absence of proper facilities, such as clean toilets, creates health risks within our community. The lack of adequate sanitation facilities hinders efforts to maintain hygiene and prevent the spread of diseases….*

(PWID/Male/41 years)

Overall, these challenges collectively highlight the barriers that the PWIDs in the community face, emphasizing the need for improved access to affordable healthcare services, adequate medical resources, and essential facilities. There is also the need for systemic improvements, policy interventions, and community initiatives to address these issues and ensure equitable access to quality healthcare services.

### 3.3. Community Approaches to Disease Prevention and Management

The approaches the PWID community used to prevent and manage diseases despite their barriers to accessing healthcare were highlighted in the analysis. These included:

“Cleaning environment”;
“Drinking clean water”;
“Sleeping under nets”;
“Using medication”;
“Resting well”;
“Sanitation and sanitization”;
“Creating awareness”;
“Avoiding risky behaviours”;
“Avoiding public toilets”.

With emphasis on “*cleaning environment*”, participants recognized the importance of maintaining a clean environment in preventing the spread of diseases. This suggests that they understand the connection between cleanliness and health and may be engaged in practices that promote a healthier living environment. Thus:

*Cleaning my environment is important for well-being and preventing disease. When we keep a clean environment, we create a safer and healthier space for ourselves and our community. It is not just about tidying up our surroundings; it is about taking responsibility for our health and preventing the spread of diseases. By cleaning our environment, we reduce the presence of disease-causing agents such as germs and stagnant water, that breeds mosquitoes.*

(PWID/Female/35 years)

Describing further their preventive measures, we deduced that, despite the understanding of the importance of sleeping under nets to prevent mosquito-borne diseases, there are deficiencies in implementing this preventive measure within the community. The lack of access to treated mosquito nets and inconsistent usage among households puts the entire community at risk of mosquito bites and the transmission of diseases. This highlights the need to address these gaps by ensuring universal access to mosquito nets and promoting consistent usage to effectively protect the community from these preventable diseases. Precisely, a participant said:

*Even though I understand the importance of sleeping under nets to prevent mosquito diseases like malaria, many of us do not still have mosquito nets in our community. Many of us lack access to treated mosquito nets, and others who have them do not use them always. This puts us in danger of mosquito bites and malaria… It is important that everyone has access to and be encouraged always use mosquito nets to protect ourselves from these preventable diseases.*

(PWID/Male/45 years)

As highlighted by participants, medication use is another preventive or management technique for diseases in the community. Although using medication is crucial for managing diseases such as malaria and typhoid, there are shortfalls in terms of availability and proper usage within the community. Some individuals may face challenges in accessing affordable and high-quality medication, while others may not adhere to the prescribed dosage or complete the full treatment course. These gaps in medication usage can result in inadequate disease management and the emergence of drug-resistant strains. Therefore, it is necessary to address these gaps by ensuring accessible and affordable medications and by educating the community on the significance of proper medication use for effective disease management.

Succinctly described:

*…We often use medications to manage diseases. As a matter of fact, using medication is essential for managing various diseases in our community such as malaria and typhoid, but then we do not have these medications always available. There is also the problem of proper usage within the community. Some people do not have easy access to quality and affordable medications, and others may not follow the prescribed dosage or complete the full dosage recommended. These challenges cause inadequate management of diseases and may lead to more stubborn ones such that drugs may not work against them again… It is important to help the community to ensure access to affordable and effective medications, and educate the community on the importance of proper medication use….*

(PWID/Male/33 years)

Participants also highlighted the issue of resting well and awareness creation in the statements below:

*Resting well is good for maintaining our health and helping the immune system, which is key to fighting diseases such as hypertension and diabetes in our community. On the other hand, the fast-paced nature of our lives and the demands of work and other responsibilities often prevent us from getting adequate rest. This problem can make hypertension worse and lead to other complications… It will be good for our people to realize the importance of rest and self-care practices within our community to better manage and prevent these diseases.*

(PWID/Female/28 years)

And also:

*Creating awareness about diseases and their preventive measures is an important aspect of teaching our community how to maintain healthy behaviours. The problem is that this awareness is not steady and adequate. Our people lack knowledge about the causes, symptoms, and preventive measures of diseases such as malaria, diabetes STIs, and hypertension. Some even think that the toilet disease is gotten from the toilet. This is the issue as they may not know how to adequately prevent and manage the conditions….*

(PWID/Male/49 years)

These two statements imply that there are several gaps in the preventive and management techniques of diseases within the PWID community, although they know that rest and awareness are necessary to prevent and manage those diseases. These gaps include a lack of prioritization of rest and self-care practices. These gaps have significant implications for the overall health and well-being of the community. Neglecting the importance of rest and self-care practices can contribute to the worsening of chronic conditions and increase the risk of complications. To address these implications, it is crucial to bridge the gaps in preventive and management techniques. This includes emphasizing the importance of rest and self-care practices, as well as creating awareness within the community to help the community better manage and prevent those diseases.

### 3.4. Health Information Needs

Health Information Needs also emerged as a specific theme with deeper insights that revealed PWID community preferred sources and channels for health information as well as needed resources and health initiatives to improve community health. Participants reflected on their health information needs. Highlights from participants showed a critical need for access to reliable and comprehensive health information to effectively manage and prevent prevalent diseases within their community. They expressed frustration over the limited availability and inadequate nature of the health information they currently receive. This lack of information hinders their ability to make informed decisions about their health and take appropriate preventive measures. These were reflected in participants’ narratives as follows:

*We feel a deep frustration with the limited availability of health information in our community. It is inadequate, leaving us without the knowledge we need to protect ourselves and our loved ones from preventable illnesses.*

(PWID/Male/28 years)

*Lack of access to reliable health information leaves us vulnerable and uncertain. We are unable to take proactive measures to prevent diseases, relying on guesswork rather than informed decision-making.*

(PWID/Male/20 years)

*Our ability to safeguard our health is compromised by the lack of reliable health information. We desire access to comprehensive resources that would equip us with the knowledge to make informed decisions and embrace preventive measures.*

(PWID/Female/33 years)

*When we think about what we really need in terms of health information, it becomes clear that we are in desperate need of reliable and complete resources. The diseases that are all around us require us to be proactive, but the problem is, we are left hanging with limited and inadequate information. It is like we are in the dark, not knowing what steps to take to protect ourselves. We want a system that gives us all the right knowledge, so we can make smart choices, take preventive action, and keep ourselves healthy. Closing this information gap is key to empowering each and every one of us in the community to live healthier lives and fight against these prevalent diseases that keep knocking us down.*

(PWID/Male/55 years)

The statements above reflect important implications regarding the community’s health information needs. Firstly, the participants highlight the critical need for access to reliable and comprehensive health information to effectively manage and prevent prevalent diseases within their community. This implies that, without such access, individuals may lack the necessary knowledge to make informed decisions about their health and take appropriate preventive measures. As a result, they may face increased vulnerability to diseases and struggle to effectively manage their well-being.

Additionally, the participants expressed frustration over the limited availability and inadequate nature of the health information they currently received. This frustration suggested that existing information sources were falling short in meeting the community’s needs, leaving them feeling uninformed and uncertain about the necessary steps needed to protect themselves. The implication here is that there is a pressing need to improve the quality and accessibility of health information. By doing so, these individuals can be better equipped to make informed choices about their health, ultimately leading to improved disease prevention and management. By ensuring access to reliable and comprehensive information, the PWIDs can be empowered to make informed decisions, take proactive measures, and ultimately enhance their overall health outcomes.

Delving deeper into their health information needs, a desire for trusted, easy-to-understand, and accessible resources was uncovered. They expressed a preference for health information presented in video formats, written articles, and those presented through outreach programs. These formats allow for visual demonstrations, clear explanations, and interactive discussions, making it easier for them to comprehend and retain important health information. By catering to these preferred formats, the information needs of the community can be met effectively and empower the PWIDs to make informed decisions about their health.

Accordingly:

*I find that health information presented in video formats is more engaging and easier to follow. Seeing visual demonstrations and explanations helps me understand complex health issues and remember important details.*

(PWID/Male/19 years)

*Written articles are my go-to source for health information. They provide a comprehensive and detailed explanation of various topics, enabling me to read at my own pace and refer back to the information whenever needed.*

(PWID/Male/39 years)

*Outreach programs have been helpful in delivering health information directly to the community. They offer interactive sessions where I can ask questions, participate in discussions, and receive guidance, making it easier for me to absorb and apply the information to my own health practices.*

(PWID/Male/47 years)

Another was of the view that:

*Community outreach is beneficial because they bring health information directly to our neighbourhood. It is comforting to have experts come to us, offering interactive sessions, discussions, and distributing informative materials that are tailored to our needs….*

(PWID/Female/27 years)

They also disclosed other multiple channels that they found beneficial in accessing health information. These include the internet, through health workers, social media, and hospitals. They believed that these channels were trustworthy and offered easy-to-understand and reliable health information. Internet platforms emerged as one of these preferred channels as participants believed it provides convenient access to a wide range of health resources and articles that can be accessed anytime, anywhere.

*I find the internet to be a valuable channel for accessing health information that I can trust and easily understand….*

(PWID/Male/22 years)

Health workers, including doctors and nurses, were also recognized as trusted sources of information, offering expertise and guidance tailored to individual needs. Regarding this, a participant opined:

*For me, health workers, such as doctors and nurses, are my go-to sources for trusted and reliable health information. They have the knowledge and experience to provide personalized advice, clarify doubts, and explain complex medical issues in a way that is easy for me to understand.*

(PWID/Male/41 years)

Additionally, social media platforms were acknowledged as influential channels for health information, allowing for the dissemination of credible and accessible content. Accordingly:

*Social media platforms have become a convenient and easy channel for health information. I appreciate following good health organizations and professionals who share informative posts in pictures, texts and videos that are both reliable and easy to understand.*

(PWID/Male/25 years)

Lastly, hospitals were seen as a reliable channel that provides comprehensive health information.

*Hospitals serve as a reliable source of health information. They offer different kinds of information that cover a wide range of health topics. I feel confident in the correctness and importance of the information they provide.*

(PWID/Female/37 years)

Overall, the preference for the internet implies that these PWIDs value the convenience and accessibility of online platforms in obtaining health information. It also suggests a reliance on digital resources to gather trusted and easily understandable information at their own pace and convenience. The mention of community outreaches suggests a desire for localized and community-based health information dissemination, implying recognition of the importance of face-to-face interactions, engagement, and tailored information that specifically addresses the needs and concerns of the local community. The preference for health workers indicates a level of trust and confidence in the expertise and guidance of healthcare professionals. It also means that the participants value the personalized advice and explanations provided by doctors, nurses, and other healthcare practitioners, considering them as reliable sources of accurate and understandable health information. The social media mentioned highlights the influence and prevalence of these platforms in shaping health information consumption. It implies recognition of the potential benefits of engaging with reputable health organizations and professionals through social media channels, where information can be easily shared, disseminated, and accessed by a broader audience. Lastly, their preference for hospitals as a source of health information suggests a perception of credibility and trust associated with healthcare institutions. It also suggests that individuals view hospitals as authoritative sources that provide reliable and comprehensive information, reinforcing the importance of reliable sources in acquiring accurate and understandable health information.

### 3.5. Essential Resources and Preferred Health Initiatives to Enhance Community Health Awareness

As co-creators and co-researchers in improving and shaping the health of PWIDs through a needs assessment, PWIDs were engaged to share their needed resources, preferred initiatives, campaigns, and health programs to improve health awareness in their communities. Among this PWID community, several options emerged as resources, initiatives, campaigns, and programs needed to address healthcare gaps and improve their overall wellbeing. These included:

“Free health care”;
“Mosquito nets”;
“Access to clean water”;
“Feeding, available and affordable medication”;
“Adequate financial resources (funds) and feeding”;
“Health outreaches”;
“Sanitation and hygiene”;
“Creating awareness”;
“Regular medical check-ups”.

It was inferred that access to free healthcare services was crucial to ensure affordability and equitable access to medical treatment. “*In this place, we need free health care… The government and other people should help us. We are human beings too*” (PWID/Male/31 years).

Mosquito nets were considered necessary to protect against mosquito-borne diseases, particularly malaria. They noted that the availability of clean and safe drinking water was essential for maintaining good health and preventing water-borne illnesses.

*Mosquitoes are too many here especially during rainy season and if mosquito bites you, you get malaria. We need mosquito nets to protect us from mosquito bites and malaria. We are also in need of clean water to drink so we don’t get sick.*

(PWID/Female/20 years)

Feeding, adequate medication supply, and affordability were reckoned as important to effectively manage and treat various diseases. Relating to sufficient funds, these were needed to support healthcare services, provide essential medications, and eat. Thus, “*…Buying medicine is expensive. Please, let us be given medicines…It is possible that one may not have money to buy drugs when they are sick…We do not have money, no good job… No good food…How can we survive?… We need money. We need medicine and food*” (PWID/Male/24 years).

Health outreaches and community programs play a significant role in raising awareness, providing education and delivering healthcare services to underserved areas. Thus, the participants said they needed these. “*…Many of us here will learn if we can have these NGOs visit us to teach us some important things…They should not look away from our side. …We need it*” (PWID/Female/28 years).

Proper sanitation facilities, hygiene practices, and awareness programs were also considered vital to prevent the spread of diseases. Creating awareness about preventive measures, disease management, and healthy behaviors is crucial for promoting overall well-being. “*This lifestyle is not easy. We need proper hygiene… More awareness is needed for some people here to understand hygiene to be healthy and other things…like managing any disease, things to do and not to do and being health generally…*” (PWID/Female/35 years). Additionally, regular medical check-ups were emphasized to detect and manage diseases at an early stage. “*…They say that early detection saves life but how can you save your life if you do not get regular check up to detect any disease early…?*” (PWID/Female/34 years).

## 4. Conclusions and Recommendations

People who inject drugs (PWIDs) are at severe risk of various disease conditions and health issues, resulting from their unsafe practices, drug effect, and the environment within which their activities take place. To effectively address the health challenges faced by the PWIDs, it is essential to strengthen outreach efforts and health education campaigns. Regular health education initiatives should be conducted, focusing on prevalent diseases, their causes, prevention strategies, and available treatment options. These campaigns must deliver clear and accurate information tailored to the specific health concerns of the community. Increasing the frequency and reach of these health outreaches is also crucial. These outreach programs should offer a wide range of services, including harm reduction education, access to clean needles, diabetes, hypertension, infections, HIV and hepatitis testing, mental health support, and referrals to treatment facilities. Collaborating with local community-based organizations can significantly enhance the effectiveness and reach of these outreach activities.

Empowering community health workers is another vital strategy. By training and equipping them with the necessary knowledge and resources, these workers can serve as reliable sources of health information within the community. They can offer personalized guidance, answer questions, and direct individuals to appropriate resources, ensuring that health information is both accessible and relevant to those in need.

Improving access to free or affordable healthcare is critical to addressing the health needs of the PWID community. Policies and initiatives should be implemented to expand existing healthcare programs, establish mobile health clinics, and collaborate with community-based organizations. This would ensure that essential medical services are accessible to all, regardless of their financial situation.

To further enhance health outcomes, the development of comprehensive and easily understandable health information materials is essential. These materials should address the specific needs and concerns of the PWIDs and be available in various formats, such as videos, written articles, and interactive online platforms. This approach caters to different preferences and literacy levels, ensuring that health information is accessible to all.

Improving access to reliable internet and technology is another important aspect of empowering the PWID community. Initiatives that provide affordable or free internet services in community centers or specific locations can help bridge gaps in health information needs. By ensuring that individuals have access to accurate and up-to-date health information online, they can make informed decisions about their health and well-being.

Enhancing funding for healthcare services that cater to the needs of the PWID community is crucial for sustaining these efforts. Advocacy for increased funding should involve engaging with policymakers, local authorities, and healthcare organizations to secure resources for the provision of medications, rehabilitation services, and mental health support for PWIDs.

Promoting health education and awareness through targeted campaigns is also critical. These campaigns should raise awareness about the risks associated with drug use, harm reduction strategies, and available healthcare resources. By delivering these campaigns through various channels, including community workshops, peer-led initiatives, and social media platforms, they can effectively reach and engage the PWID community.

Finally, advocating for improved infrastructure and facilities is necessary for creating a supportive environment for the PWID community. Establishing healthcare facilities, including clinics and treatment centers, near the community, as well as providing clean water sources, sanitation facilities, and safe living environments are essential steps in maintaining good health and preventing the spread of diseases. Implementing these recommendations would address the identified challenges and health information gaps, ultimately improving the health outcomes and well-being of the PWIDs.

## 5. Limitations

It is important to acknowledge the limitations and potential biases inherent in this study. Firstly, it is crucial to note that qualitative research aims for in-depth understanding rather than statistical representativeness. Therefore, the findings of this study may not be generalized to all people who inject drugs (PWIDs) in Nigeria. The sample of participants was selected through purposive and snowball sampling techniques, which may introduce certain biases and limitations in terms of the diversity and representativeness of the population under study. As a result, caution should be exercised when attempting to apply the findings to a broader population.

Furthermore, it is important to consider the influence of social desirability bias and potential underreporting of sensitive information. PWIDs may feel stigmatized or fear legal repercussions, which could lead to a reluctance to disclose certain information or provide socially desirable responses. Despite efforts to establish a safe and confidential environment, participants may still feel apprehensive about sharing sensitive details related to their drug use or health-related experiences. This could potentially impact on the accuracy and completeness of the data collected.

To mitigate these limitations, the researchers employed techniques such as establishing rapport, ensuring privacy and confidentiality, and were skilled interviewers sensitive to the unique needs and concerns of the participants. These measures aimed to create a supportive environment that encouraged participants to share their experiences openly. Nevertheless, it is important to interpret the findings with caution, considering the potential biases and limitations, as well as the nature of the study population.

By acknowledging these limitations and biases, researchers and readers can better understand the contextual and interpretive nature of the findings. While the study provides valuable insights into the disease condition and health information needs among PWIDs in Plateau State, Nigeria, further research with larger and more diverse samples is recommended to enhance the generalizability of the findings and to capture a wider range of perspectives and experiences within this population. We also recommend such studies to explore the onset of drug usage and predisposing factors to such lifestyles by employing key techniques such as life history.

## Figures and Tables

**Table 1:** Participants’ demographic information.

Variable	Frequency (N = 71)	Percentage (100%)
**Gender**		
Male	43	60.6
Female	28	39.4
**Age**		
19–25 years	19	26.8
26–35 years	42	59.2
36–45 years	3	4.2
46–55 years	5	7.0
55 yrs and above	2	2.8
**Marital Status**		
Married	24	33.8
Single	47	66.2
**Highest Level of Education**		
Primary	6	8.5
Secondary	37	39.4
Tertiary	28	39.4
**Occupation**		
Artisans/Self-Employed	31	43.7
Trader	13	18.3
Civil Servant	1	1.4
Student	10	14.1
Employee in a private firm	3	4.2
Unemployed	13	18.3
**Average Monthly Income**		
Less than 1000	18	25.4
1000 to 10,000	20	28.2
10,001 to 20,000	11	15.5
20,001 to 30,000	13	18.3
30,001 to 40,000	5	7.0
40,000 and above	4	5.6

## Data Availability

Data are available upon reasonable request.
